# C_2_HEST score for atrial fibrillation risk prediction models: a Diagnostic Accuracy Tests meta-analysis

**DOI:** 10.1186/s43044-021-00230-0

**Published:** 2021-12-04

**Authors:** Habib Haybar, Kimia Shirbandi, Fakher Rahim

**Affiliations:** 1 Atherosclerosis Research Center, Ahvaz Jundishapur University of Medical Sciences, Ahvaz, Iran; 2 Ahvaz Jundishapur University of Medical Sciences, Ahvaz, Iran; 3 Thalassemia and Hemoglobinopathy Research Center, Research Institute of Health, Ahvaz Jundishapur University of Medical Sciences, Ahvaz, Iran

**Keywords:** C_2_HEST score, Meta-analysis, Atrial fibrillation (AF), Risk

## Abstract

**Background:**

This meta-analysis aimed to assess the value of the C_2_HEST score to facilitate population screening and detection of AF risk in millions of populations and validate risk scores and their composition and discriminatory power for identifying people at high or low risk of AF. We searched major indexing databases, including Pubmed/Medline, ISI web of science, Scopus, Embase, and Cochrane central, using (“C2HEST” OR “risk scoring system” OR “risk score”) AND (“atrial fibrillation (AF)” OR “atrial flutter” OR “tachycardia, supraventricular” OR “heart atrium flutter”) without any language, study region or study type restrictions between 1990 and 2021 years. Analyses were done using Meta-DiSc. The title and abstract screening were conducted by two independent investigators.

**Results:**

Totally 679 records were found through the initial search, of which ultimately, nine articles were included in the qualitative and quantitative analyses. The risk of AF accompanied every one-point increase of C_2_HEST score (OR 1.03, 95% CI 1.01–1.05, *p* < 0.00001), with a high heterogeneity across studies (*I*^*2*^ = 100%). The SROC for C_2_HEST score in the prediction of AF showed that the overall area under the curve (AUC) was 0.91 (95% CI 0.85–0.96), AUC in Asian population was 0.87 (95% CI: 0.78–0.95) versus non-Asian 0.95 (95% CI 0.91–0.99), and in general population was 0.92 (95% CI 0.85–0.99) versus those with chronic conditions 0.83 (95% CI 0.71–0.95), respectively.

**Conclusions:**

The results of this research support the idea that this quick score has the opportunity for use as a risk assessment in patients' AF screening strategies.

**Supplementary Information:**

The online version contains supplementary material available at 10.1186/s43044-021-00230-0.

## Abstract (articlehighlights)

- Use of C2HEST score to predict Atrial Fibrillation.
- For identifying people at high or low risk of Atrial Fibrillation.
- Assessment the value of the C2HEST score.

**Supplementary Information:**

The online version contains supplementary material available at 10.1186/s43044-021-00230-0.

## Background

Atrial fibrillation (AF) is a common type of arrhythmia or irregular heartbeat, which is defined as a supraventricular tachyarrhythmia characterized by uncoordinated atrial activity and subsequent mechanical atrial failure [1]. AF by disrupting heart function and increasing stroke risk accounts a significant source of mortality [2]. It was demonstrated AF affects about 1% of people under 60 years and 8% of people over 80-year [3], and approximately 2.3 million people in North America and 4.5 million in the EU Member States suffer from this disease [4–6]. Surveys such as conducted by Kannel et al. have shown that about a third of hospitalizations for rhythm disorders are due to this disorder, which has grown by 66% in the last 20 years [7].

## Main text

Several factors contribute to this increasing, including population aging, increased prevalence of chronic heart disease (CHD), and *improving* diagnostic ability due to the *advancing* of technologies and equipment [8]. Therapeutic strategies in managing AF are based on interventions that control heart rate or rhythm [9]. Thromboembolism is one of the life-threatening adverse events in AF that, for preventing it, anticoagulant therapy is essential. However, treatment and management of patients with AF should be based on the disease’s type [10].

To predict incident AF, numerous risk scores considering instrumental and laboratory factors have been established so far [11–13]. By predicting AF risk in a timely manner, especially using various risk scoring systems, it is possible to control the disease and prevent its complications by using preventive treatment methods [14]. The C_2_HEST score (C_2_, coronary artery disease or chronic obstructive pulmonary disease [1 point each]; H, hypertension [1 point]; E, elderly [age ≥ 75 years, 2 points]; S, systolic heart failure [2 points]; T, thyroid disease [hyperthyroidism, 1 point]), the latest, easy-to-use and most straightforward risk scoring system was initially introduced and validated through large population-based cohorts of healthy individuals and patients with chronic diseases [15–17].

Therefore, this systematic review and meta-analysis aimed to assess the value of the C_2_HEST score to facilitate population screening and detection of AF risk in over millions of general populations and those with chronic diseases, and the validation of risk scores and their composition and discriminatory power for identifying people at high or low risk of AF.

## Methods

This systematic review and meta-analysis were conducted according to the Meta-analyses Of Observational Studies in Epidemiology (MOOSE) [18] and Preferred Reporting Items for Systematic reviews and Meta-Analyses (PRISMA) [19] and SEDATE (Synthesizing Evidence from Diagnostic Accuracy Tests) guidelines [20].

### Search strategy

We searched major indexing databases, including Pubmed/Medline, ISI web of science (WOS), Scopus, Embase, and Cochrane central, using (“C2HEST” OR “riskscoring system” OR “risk score”) AND (“atrial fibrillation (AF)” OR “atrial flutter” OR “tachycardia, supraventricular” OR “heart atrium flutter”) without any language, study region, or study type restrictions between 1990 and 2021 years.

### Inclusion criteria

Criteria for selecting studies were as follows, considering individuals from either the general population or those with chronic diseases susceptible to AF occurrence, and larger prospective, national, population-based studies using C_2_HEST score for predicting the risk of AF. Studies that evaluated the C_2_HEST score in other heart disorders or investigated other scoring systems in AF were excluded.

### Study selections

After removing duplicated studies, two authors (HH and FR) independently screened titles and abstracts of potential papers considering pre-defined inclusion and exclusion criteria. Any disagreements were resolved by either re-evaluating the source article or consulting a third author (ME). Two independent investigators conducted the title and abstract screening.

### Data extraction

Information, including author’s name, publication year, country, age, sample size, and study design.

### Methodological quality assessment

Two reviewers (HH and FR) performed the quality assessment of included studies using the Newcastle–Ottawa Scale (NOS) and the Quality Assessment of Diagnostic Accuracy Studies (QUADAS-2) tools. Disagreements were resolved by either discussing or re-evaluating the original article with a third reviewer (ME).

### Ethical consideration

Ethical committee approval and informed consent were not essential due to working on previously published studies.

### Statistical analysis

We retrieved the odds ratio (OR) with 95% confidence interval (CI) from the eligible studies and calculated summary OR (SOR) with the random-effects or fixed-effect models depending on the level of heterogeneity to evaluate the association of C_2_HEST score with the risk of AF [21]. Afterward, we measured heterogeneity across studies using Cochran’s Q statistics and *I*^*2*^ test. When *I*^2^ values (more than 50%) showed a high heterogeneity sensitivity and subgroup analyses were performed to discover the heterogeneity source. A hierarchical receiver-operating characteristic summary (HSROC) curve and a summary receiver operating characteristic (SROC) curve have been mounted. All experiments were viewed with the HSROC curve as a circle and plotted. The area under the curve (AUC) was computed to determine the diagnostic precision. Approaches 1.0 to the AUC would mean outstanding results, and impaired performance would be suggested if it approaches 0.5. Among numerous subgroups, the 95% CI of the AUC was compared. When the sensitivity and specificity were directly unavailable, they were calculated according to the following formulas: sensitivity = TP/(TP + FN) and specificity = TN/(FP + TN). Publication bias was measured using Deeks’ regression test [22]. Subgroup analysis was done according to the NOS assessment, C2HEST score for AF prediction, Ethnicity, General population, and Chronic conditions. The analysis was conducted using version 1.4 of the Meta-DiSc software (https://meta-disc.software.informer.com/1.4/) [23] and Revman 5.3.

## Results

### Search results

**Fig. 1:**
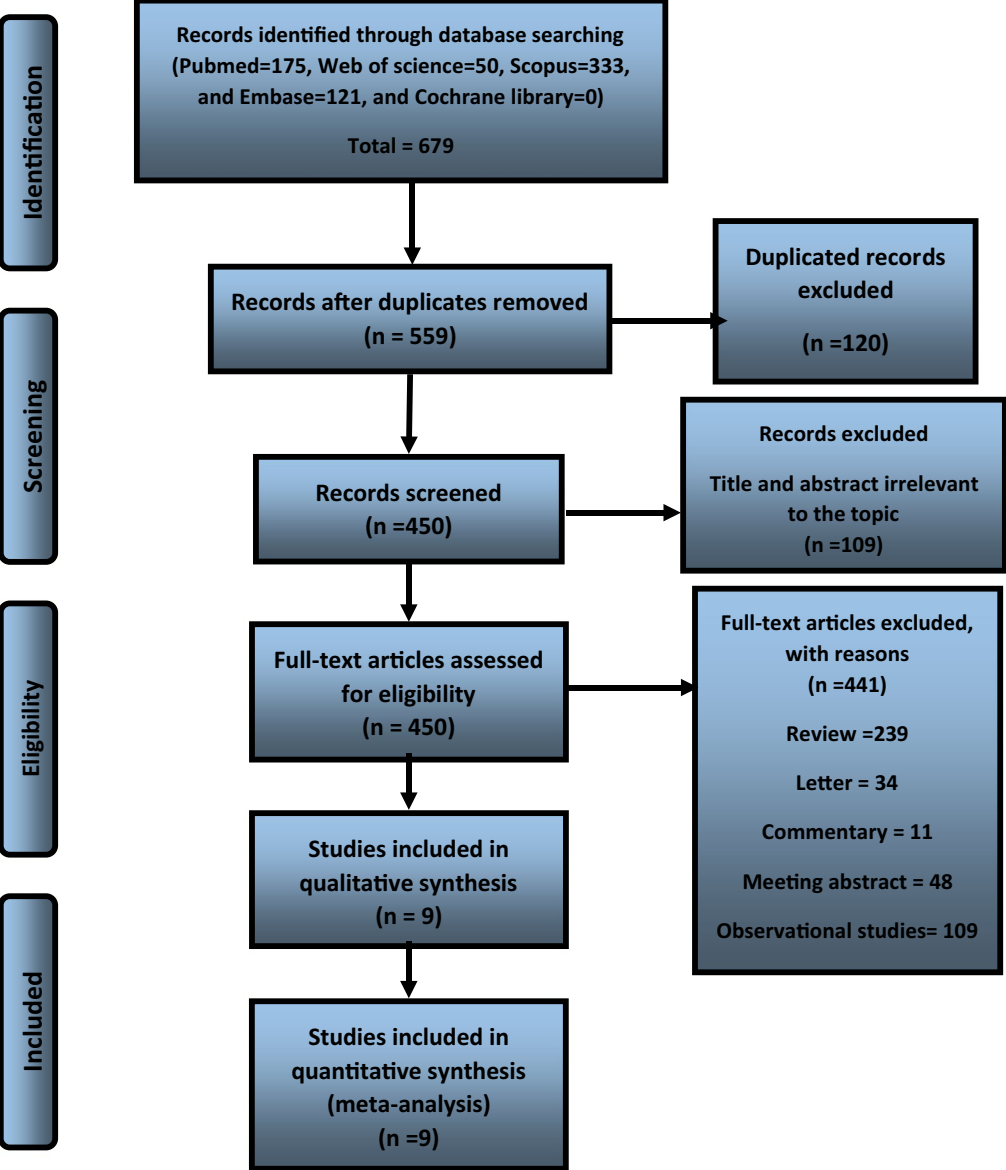
Flow diagram of the selection process

Totally 679 records were found through the initial search. Of 679 articles, 120 duplicated studies were found, and 109 were omitted due to irrelevant titles and abstracts. The rest 450 were entered the full-text screening, of which 441 were excluded due to pre-defined inclusion criteria. Ultimately, nine articles were included in the qualitative and quantitative analyses (Fig. 1).

### Study characteristics

There were 6,293,676 general population and 2,741,896 patients in the nine eligible studies, of which 310,649 cases developed AF. Five studies sampled individuals from the general population [14, 15, 24–26], while the rest four included patients with chronic conditions [16, 27–29] (Table 1). In general population, the average age of the included participants was from 34.0 to 62.5 years (females took up 10.8–58%); whereas, in the chronic condition population, the average age of the included participants was from 52.6 to 70.8 years (females took up 47–54.7%). The average C_2_HEST score of the included participants was from 0.32 to 4.7. The majority of the included participants had hypertension among various comorbidities, ranging from 15.28 to 91.99%.

**Table 1:** Patient characteristics

Study	Country	Group	Population	Mean follow-up, years (SD)	Age (years)	Female (%)	HTN, n (%)	HF, n (%)	DM, n (%)	CAD, n (%)	COPD, n (%)	AF, n (%)	Mean C_2_HEST
Guo et al. [30]	China	HHS	209,274 GP	NA	34 ± 11.30	10.6%	31,987 (15.28%)	3286 (1.57%)	7620 (3.64%)	6146 (2.94%)	68,335 (32.66%)	491 (0.23)	1 ± 0.47
Hu and Lin [24]	Taiwan	National cohort	692,691 GP	10.9 ± 2.74	41.3 ± 16.3	49.4%	99,794 (14.4%)	4120 (0.59%)	NA	43,301 (6.25%)	25,379 (3.66%)	209 (0.03)	0.32 ± 0.79
Khurshid et al. [26]	USA	EHR-AF	4,508,180 GP	3.1 9 ± 1.98	62.5 ± 10.9	56.3%	2,375,811 (52.7%)	166,803 (3.7%)	978,275 (21.7%)	545,490 (12.1%)	NA	283,783 (6.3)	NA
Liag et al. [29]	China	TOPCAT	2202 CD	3.89 ± 0.98	67.01 ± 9.44	54.7%	2024 (91.9%)	NA	752 (34.2%)	NA	231 (10.5%)	130 (5.9)	NA
Lip et al. [16]	Denmark	Danish Cohort Study	2,499,235 CD	4.92 ± 1.18	65.08 ± 10.2	52.9%	611,117 (24.45%)	103,769 (4.15%)	142,507 (5.70%)	229,054 (9.16%)	108,692 (4.35%)	132,012 (5.2)	1.16 ± 1.01
Hu and Lin [27]	Taiwan	NHIRD	4601 CD	10.9 ± 2.74	62.6 ± 14.3	50.2%	4174 (90.7%)	1029 (22.4%)	2351 (51.1%)	2082 (45.3%)	874 (19.0%)	209 (4.5)	2.33 ± 1.55
Hulme et al. [25]	USA	RPDR	412,085 GP	4.92 ± 1.18	61.0 ± 11.0	58%	115,384 (28%)	12,775 (3.1%)	38,736 (9.4%)	38,324 (9.3%)	NA	29,035 (7.04)	NA
Li et al. [28]	France	FNS	240,459 CD	7.9 ± 11.5	70.8 ± 15.7	47%	141,045 (90.7%)	33,162 (14.7%)	50,977 (22.5%)	39,652 (17.5%)	35,320 (15.6%)	66,811 (27.7)	4.7 ± 1.99
Li et al. [15]	China	CYID	471,446 GP	4.1 ± 3.5	56.1 ± 9.3	46%	143,168 (31.7%)	5515 (1.2%)	37,372 (8.3%)	9946 (2.2%)	44,470 (9.9%)	921 (0.19)	0.75 ± 0.56

*HTN* hypertension, *HF* heart failure, *DM* diabetes mellitus, *CAD* coronary artery disease, *COPD* chronic obstructive pulmonary disease, *LAD* left atrial diameter, *LVEF* left ventricular ejection fraction, *ACEIs* angiotensin converting enzyme inhibitors, *ARBs* angiotensin receptor blockers, *CCB* calcium channel blockers, *GP* general population, *CD* chronic diseases, *RPDR* Partners HealthCare System Research Patient Data Registry, *FNS* A French Nationwide Study, *CYID* Chinese Yunnan Insurance Database, *AF* atrial fibrillation

### Meta-analysis

**Fig. 2:**
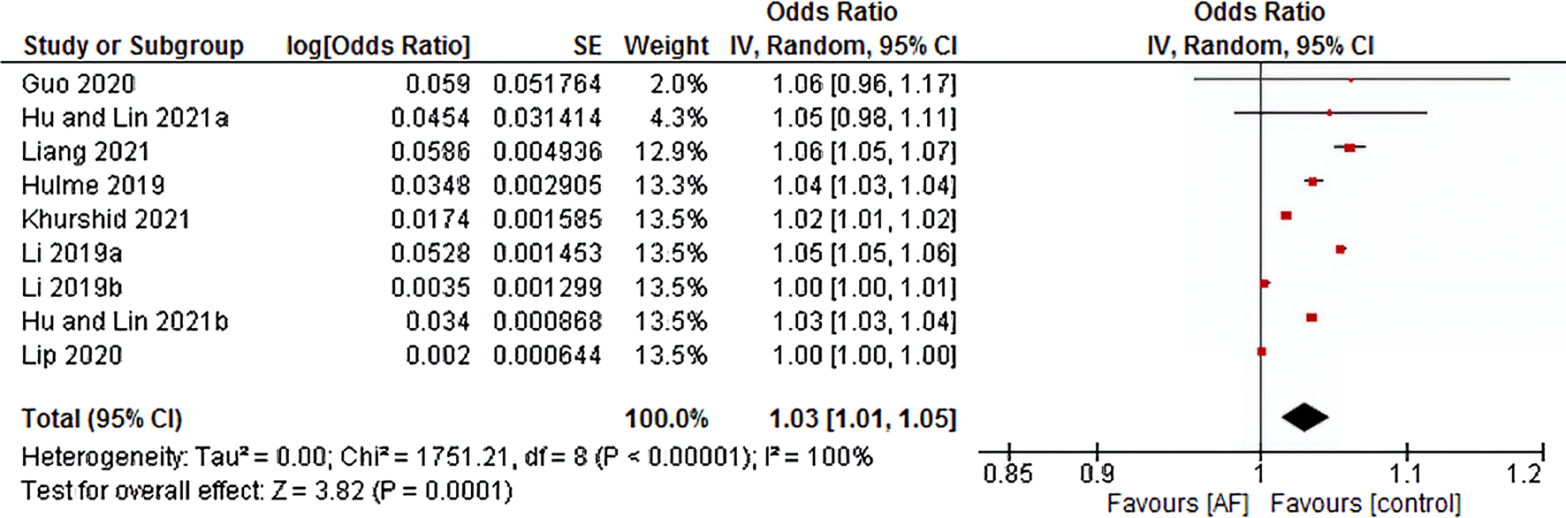
Forest plot of the association between C_2_HEST score and atrial fibrillation. *CI* confidence interval

Five of nine included studies were rated as high-quality according to the NOS assessment, and the rest four were rated as medium quality studies (Additional file 1: Table S1). Our data have shown that the risk of AF is accompanied 3% by every one-point increase of C_2_HEST score (OR 1.03, 95% CI 1.01–1.05, *p* < 0.00001), with a high heterogeneity across studies (*I*^*2*^ = 100%) (Fig. 2). Six of the nine included studies observed the performance of C_2_HEST score for AF prediction [15, 16, 24, 27, 28, 30], of which four of them rated as medium quality studies using the QUADAS-2 framework (Additional file 2: Table S2). We retrieved the sensitivity, specificity, and accuracy test result in the nine included studies. The sensitivity of C_2_HEST score in predicting AF was from 66.9 to 94.8%, specificity from 51.58 to 98.18%, and accuracy from 59.21 to 78.95% (Additional file 3: Table S3).

Our pooled analysis consequently revealed that the C_2_HEST score had reasonably higher sensitivity in predicting the AF, especially in the Asian comparison to non-Asian population (Table 2).

**Table 2:** C_2_HEST score accuracy estimates from the stratified bivariate regression analysis

Study characteristics (no. of studies)	Pooled sensitivity (95% CI)	Pooled specificity (95% CI)	AUC	*P* value*
*Ethnicity*				0.01*
Asian (5)	0.79 [0.67–0.89]	0.71 [0.52–0.98]	0.87 [0.78–0.95]	
Non-Asian (4)	0.73 [0.69–0.95]	0.95 [0.87–0.96]	0.95 [0.91–0.99]	
*Condition*				0.58
General population (5)	0.82 [0.78–0.95]	0.76 [0.53–0.85]	0.92 [0.85–0.99]	
Chronic conditions (4)	0.71 [0.67–0.79]	0.95 [0.87–0.98]	0.83 [0.71–0.95]	
Overall (pooled)	0.74 [0.69–0.95]	0.88 [0.52–0.98]	0.91 [0.85–0.96]	–

*AUC* area under the curve

*P value for the joint model

**Fig. 3:**
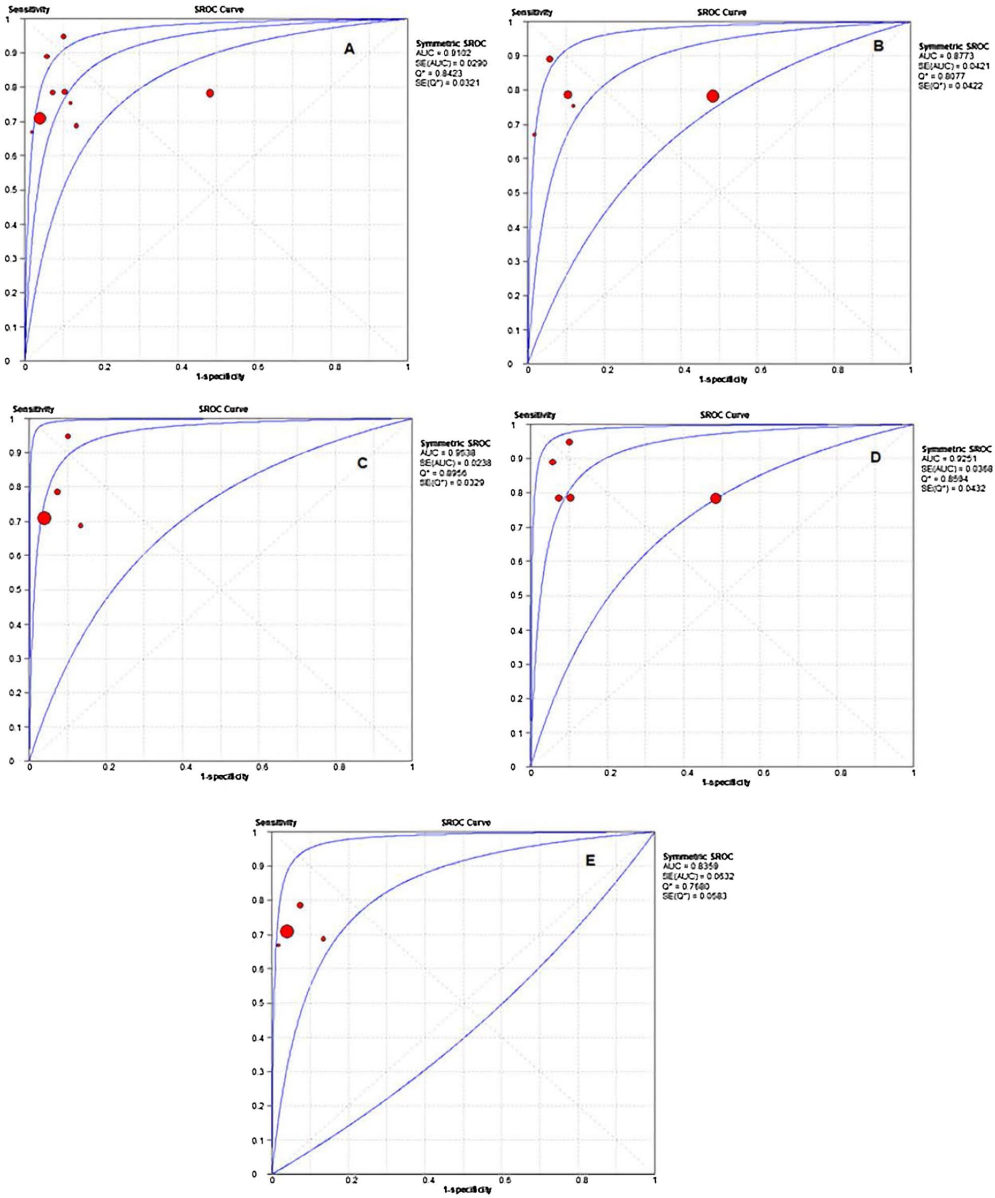
Summary receiver operating characteristic curve (SROC) analysis. The red circle symbol represents the summary estimate of sensitivity and specificity of the C_2_HEST score using a bivariate random-effects regression model. A dotted line surrounds the circle represents the 95% confidence interval. *AUC* area under the curve. **A** The overall AUC, **B** Asian, **C** non-Asian, **D** general population, and **E** chronic conditions

The SROC for C_2_HEST score in the prediction of AF showed that the overall area under the curve (AUC) was 0.91 (95% CI 0.85–0.96) (Fig. 3A), AUC in Asian population was 0.87 (95% CI 0.78–0.95) versus non-Asian 0.95 (95% CI 0.91–0.99) (Fig. 3B, C), and in general population was 0.92 (95% CI 0.85–0.99) versus those with chronic conditions 0.83 (95% CI 0.71–0.95) (Fig. 3D, E), respectively.

## Discussion

The result of this study indicates a positive association between the C_2_HEST score and the AF. The most prominent finding to emerge from the analysis is that each one-point increase in C_2_HEST score was associated with risk of AF, on the other hand, our analysis showed that none of the subgroups were significantly different in any of the measured variables except for the specificity between general population and chronic condition.

Therefore, the AUC and pooled specificity of C_2_HEST score in the general population versus those with chronic conditions were higher. Moreover, this study's pooled specificity and sensitivity are in line with those of previous studies in the 86.5–89.8 range and 75.01–78.6 range, respectively [15, 27, 28, 30]. The highest OR was observed in the studies conducted by Guo et al*.* (OR 1.6, 95% CI 0.96–1.17) and Liang et al*.* (OR 1.6, 95% CI 1.05–1.07) [29, 30], and the lowest was observed in Lip et al*.,* (OR 1, 95% CI 1–1) and Li et al*.,* reports (OR 1, 95% CI 1–1.03) [15, 16]*.* Also, Guo et al*.*, Liag et al*.*, and Hu and Lin included high-risk population with hypertension (HTN), heart failure (HF), diabetes mellitus (DM), coronary artery disease (CAD), and Chronic obstructive pulmonary disease (COPD) [27, 29, 30].

A recent study that set out to determine the usability of C_2_HEST and HATCH scores in AF prediction demonstrated that the C_2_HEST score appeared to be more predictive of AF versus HATCH score [24]. This agree with Li et al*.* findings that the C_2_HEST score can be used as a simple clinical tool to determine an individual’s probability of developing AF in Asians who do not have structural heart disease (SHD) [15]. However, the AUROC values for CHA2DS2-VASc and C_2_HEST are almost equal, meaning that there is a little difference in predictive ability [27]. In that case, white European population hospitalized with prior ischemic stroke, the C_2_HEST score performed well in predicting the risk of experiencing event AF [28].

Furthermore, a higher risk C_2_HEST score was linked to an increased risk of new onset AF. More extensive efforts for screening and diagnosing event AF may be considered for these patients [16]. In addition, the C_2_HEST score, particularly when paired with symptoms, can make a population-based screening and prevention approach for AF more feasible [30].

Detailed examination of Identifying At-Risk Patients C_2_HEST score by Li et al. has shown that in patients without AF who had a cardiac implantable electronic unit; the C_2_HEST score estimated the occurrence of sustained atrial high-rate episodes (SAHREs); consequently, patients with a C_2_HEST score of four having the greatest chance [31]. Also, in patients with heart failure and retained ejection fraction, the C_2_HEST score could forecast the likelihood of event AF, death, and hospitalization for heart failure with preserved ejection fraction (HFpEF). Its flexibility can make fast risk evaluations possible in busy clinical settings [29].

## Limitations

This study has some limitations; a patient with hypertension for 20 years is more likely to encounter AF than another with only a two-year history of hypertension. Also, the degree of compliance to treatment and the degree of control of CHEST components were not mentioned, so the drugs given can affect AF.

## Conclusions

This investigation aimed to achieve a reliable, accurate, and easy-to-performance method for predicting AF development. This study has identified that the C_2_HEST score has good performance in predicting AF and could help identify the individuals at high risk of AF in the Asian and non-Asian populations. The results of this research support the idea that this quick score can be used as a risk assessment in patients' AF screening strategies.

## Supplementary Information

**Additional file 1**. **Table S1**: Study quality of included studies based on the Newcastle-Ottawa scale.

**Additional file 2**. **Table S2**: Study quality of included studies based on the QUADAS-2 tool.

**Additional file 3**. **Table S3**: Different predictive ability of C2HEST score for AF-associated risk in Asian patients or non-Asian patients.

## Data Availability

The datasets generated and/or analyzed during the current study are available in the [Pubmed, Web of Science, Scopus, EM Base] repository.

## Abbreviations

WOS: Web of science
AF: Atrial fibrillation
CHD: Chronic heart disease
C_2_HEST score: C_2_, coronary artery disease or chronic obstructive pulmonary disease; H, hypertension; E, elderly (age ≥ 75 years); S, systolic heart failure; T, thyroid disease (hyperthyroidism)
MOOSE: Meta-analyses Of Observational Studies in Epidemiology
PRISMA: Systematic reviews and Meta-Analyses
SEDATE: Synthesizing Evidence from Diagnostic Accuracy Tests
NOS: Newcastle–Ottawa Scale
QUADAS-2: Quality Assessment of Diagnostic Accuracy Studies
OR: Odds ratio
CI: Confidence interval
SOR: Summary OR
HSROC: Hierarchical receiver-operating characteristic summary
SROC: Summary receiver operating characteristic
AUC: Area under the curve
HTN: Hypertension
HF: Heart failure
DM: Diabetes mellitus
CAD: Coronary artery disease
COPD: Chronic obstructive pulmonary disease
SHD: Structural heart disease
SAHREs: Sustained atrial high-rate episodes
HFpEF: Heart failure with preserved ejection fraction
