# Supplementary material for: C2HEST score for atrial fibrillation risk prediction models: a Diagnostic Accuracy Tests meta-analysis
Source: Egypt Heart J. 2021 Dec 4;73:104. doi: 10.1186/s43044-021-00230-0 (PMC8643379; doi:10.1186/s43044-021-00230-0)
Supplement: Supplementary file 1 — Additional file 1. Table S1: Study quality of included studies based on the Newcastle-Ottawa scale. [file 43044_2021_230_MOESM1_ESM.docx]

**Table S1:** Study quality of included studies based on the Newcastle-Ottawa scale

| **Study** | ***Selection*** | | | | **Comparability** | **Outcome** | | | **Score** |
| --- | --- | --- | --- | --- | --- | --- | --- | --- | --- |
|  | **Representativeness of the exposed cohort** | **Selection of the non-exposed cohort** | **Ascertainment of exposure** | **Demonstration that outcome of interest was not present at start of study** | **Comparability of cohorts on the basis of the design or analysis** | **Assessment of outcome** | **Was follow-up long enough for outcomes to occur** | **Adequacy of follow up of cohorts** |  |
| Guo *et al*. 2021 [1] | ★ | ★ | ★ | ★ | ★★ | ★ | ★ | ☆ | 8 |
| Hu and Lin, 2021 [2] | ★ | ★ | ☆ | ★ | ★ | ☆ | ★ | ★ | 6 |
| Khurshid *et al*. 2021 [3] | ★ | ★ | ★ | ★ | ★★ | ★ | ☆ | ☆ | 7 |
| Liag *et al*. 2021 [4] | ★ | ★ | ★ | ★ | ★★ | ★ | ☆ | ☆ | 7 |
| Lip et al. 2020 [5] | ★ | ★ | ★ | ★ | ★★ | ★ | ★ | ☆ | 8 |
| Hu and Lin, 2020 [6] | ★ | ★ | ★ | ★ | ★★ | ★ | ★ | ☆ | 8 |
| Hulme et al. 2019 [7] | ★ | ★ | ★ | ★ | ★★ | ★ | ★ | ☆ | 8 |
| Li et al. 2019 [8] | ★ | ★ | ★ | ★ | ★ | ★ | ★ | ★ | 8 |
| Li et al. 2019[9] | ★ | ★ | ☆ | ★ | ★★ | ★ | ★ | ☆ | 7 |

Q1: The exposed cohort was truly or somewhat representative? Q2: The non-exposed cohort was drawn from the same community as the exposed cohort? Q3: Exposure was ascertained by secure record or structured interview? Q4: Outcome of interest was not present at start of study? Q5: On the basis of the design or analysis cohorts had comparability? (controls for the most important factor? controls for any additional factor?) Q6: Outcome was independent blind assessment or record linkage? Q7: Follow-up was long enough for outcomes to occur? Q8: Follow-up of cohorts was adequate? A study can be awarded a maximum of one star for each numbered item within the selection and outcome categories. A maximum of two stars can be given for comparability.
