# Supplementary material for: C2HEST score for atrial fibrillation risk prediction models: a Diagnostic Accuracy Tests meta-analysis
Source: Egypt Heart J. 2021 Dec 4;73:104. doi: 10.1186/s43044-021-00230-0 (PMC8643379; doi:10.1186/s43044-021-00230-0)
Supplement: Supplementary file 2 — Additional file 2. Table S2: Study quality of included studies based on the QUADAS-2 tool. [file 43044_2021_230_MOESM2_ESM.docx]

**Table S2: Study quality of included studies based on the QUADAS-2 tool**

| **Studies** | **Risk of bias** | | | | **Applicability Concerns** | | |
| --- | --- | --- | --- | --- | --- | --- | --- |
|  | **Patient selection** | **Index text** | **Reference standard** | **Flow and timing** | **Patient selection** | **Index text** | **Reference standard** |
| Guo et al, 2021 | ☺ | 〇 | ☺ | ☺ | ☺ | ☺ | ☺ |
| Hu and Lin, 2021a | ☺ | 〇 | ☺ | ☺ | ☺ | ☺ | ☺ |
| Hu and Lin, 2021b | ☺ | ☹ | ☺ | ☺ | ☺ | ☺ | ☺ |
| Lip et al, 2019 | ☺ | ☺ | ☺ | ☺ | ☺ | ☺ | ☺ |
| Li et al, 2019a | ☺ | ☺ | ☺ | ☺ | ☺ | ☺ | ☺ |
| Li et al, 2019b | ☺ | ☹ | ☺ | ☺ | ☺ | ☺ | ☺ |

☺ Low risk 〇 unclear risk ☹ high risk
