# Supplementary material for: C2HEST score for atrial fibrillation risk prediction models: a Diagnostic Accuracy Tests meta-analysis
Source: Egypt Heart J. 2021 Dec 4;73:104. doi: 10.1186/s43044-021-00230-0 (PMC8643379; doi:10.1186/s43044-021-00230-0)
Supplement: Supplementary file 3 — Additional file 3. Table S3: Different predictive ability of C2HEST score for AF-associated risk in Asian patients or non-Asian patients. [file 43044_2021_230_MOESM3_ESM.docx]

**Table S3:** Different predictive ability of C_2_HEST score for AF-associated risk in Asian patients or non-Asian patients

| **Study ID** | **Country** | **Patients** | **AUC** | **95% CI** | **Sensitivity** | **Specificity** |
| --- | --- | --- | --- | --- | --- | --- |
| Liang et al, 2021 | Asian (China) | Heart failure with preserved ejection fraction (HFpEF) patients | 0.649 | 0.640 -0.748 | 89.67% | 94.23% |
| Khurshid et al, 2021 | Non-Asian (USA) | Individuals with complete AF | 0.683 | 0.682 - 0.684 | 78.25% | 51.58% |
| Hu and Lin, 2021b | Asian (Taiwan) | End-stage renal disease patients | 0.7895 | 0.780 - 0.812 | 94.8% | 89.8% |
| Guo et al, 2021 | Asian (China) | Population with Palpitations | 0.73 | 0.689 - 0.745 | 75.01% | 88.01% |
| Hu and Lin, 2021a | Asian (Taiwan) | Population with Palpitations | 0.5983 | 0.5412 - 0.6160 | 70.88% | 96.4% |
| Hulme et al, 2020 | Non-Asian (USA) | Population with Palpitations | 0.754 | 0.747 - 0.762 | 66.9% | 98.18% |
| Lip et al, 2019 | Non-Asian (Denmark) | Population with Palpitations | 0.5921 | 0.5412 - 0.6160 | 78.5% | 92.6% |
| Li et al, 2019a | Non-Asian (France) | Population with Palpitations | 0.734 | 0.732 - 0.736 | 68.7% | 86.5% |
| Li et al, 2019b | Asian (China) | Population with Palpitations | 0.75 | 0.730 - 0.771 | 78.6% | 89.5% |
